# Intramural Ventricular Arrhythmias: How to Crack a Hard Nut

**DOI:** 10.1007/s11886-024-02143-1

**Published:** 2024-11-27

**Authors:** Matthew Hanson, Andres Enriquez, Fermin Garcia

**Affiliations:** 1 Division of Cardiology, Queen’s University, Kingston, Ontario Canada; 2 Section of Cardiac Electrophysiology, Hospital of the University of Pennsylvania, 1 Convention Avenue, Philadelphia, Pennsylvania 19104 USA

**Keywords:** Intramural, Premature ventricular contraction, Ventricular arrhythmia, Catheter ablation, Mapping

## Abstract

**Purpose of the Review:**

Successful catheter ablation of ventricular arrhythmias depends on identifying the critical tissues that sustain the arrhythmia. Increasingly, the intramural space is being recognized as an important source of idiopathic and reentrant ventricular arrhythmias, representing a common cause of ablation failure. A systematic approach to mapping and ablating these arrhythmias is key to optimize outcomes.

**Recent Findings:**

Intramural ventricular arrhythmias are common in certain anatomical locations such as the left ventricular ostium or the interventricular septum. In these cases, mapping of the septal coronary veins provides an opportunity to explore the intramural compartment of the septum to perform activation mapping, entrainment and/or pace mapping. When an intramural arrhythmia is identified, ablation may require radiofrequency application from multiple sites, prolonged lesions, or special ablation techniques such as bipolar ablation or transvenous ethanol injection.

**Summary:**

Identification of intramural ventricular arrhythmias depends on comprehensive mapping that should include the coronary venous system, and ablation often requires advanced techniques. This paper provides a guide on when to suspect an intramural ventricular arrhythmia in the electrophysiology laboratory and how to approach mapping and ablation in these challenging cases.

## Background

Catheter ablation (CA) is a safe and effective therapy for both idiopathic and scar-related ventricular arrhythmias (VAs). Occasionally, the site of origin (focal VA) or critical isthmus (reentrant VA) may be located deep within the ventricular walls, posing significant challenges for mapping and ablation [1], and resulting in suboptimal outcomes.

An intramural origin is common in idiopathic VAs from the left ventricular (LV) ostium, accounting for at least 20% [2] in some series, and up to 40% of VAs with suspected LV summit origin when comprehensive mapping is conducted [3]. Similarly, in patients with ventricular tachycardia (VT) in the context of structural heart disease an intramural reentry can be demonstrated by simultaneous endocardial and epicardial mapping in about 1 out of 5 patients [4], with a higher prevalence in nonischemic etiology.

In recent years, advances in mapping and ablation technologies have allowed increased recognition and improved ablation success in these patients. This review outlines the main elements to suspect and diagnose an intramural VA and the potential ablation strategies to target them.

## Identification of Intramural Substrate

**Fig. 1:**
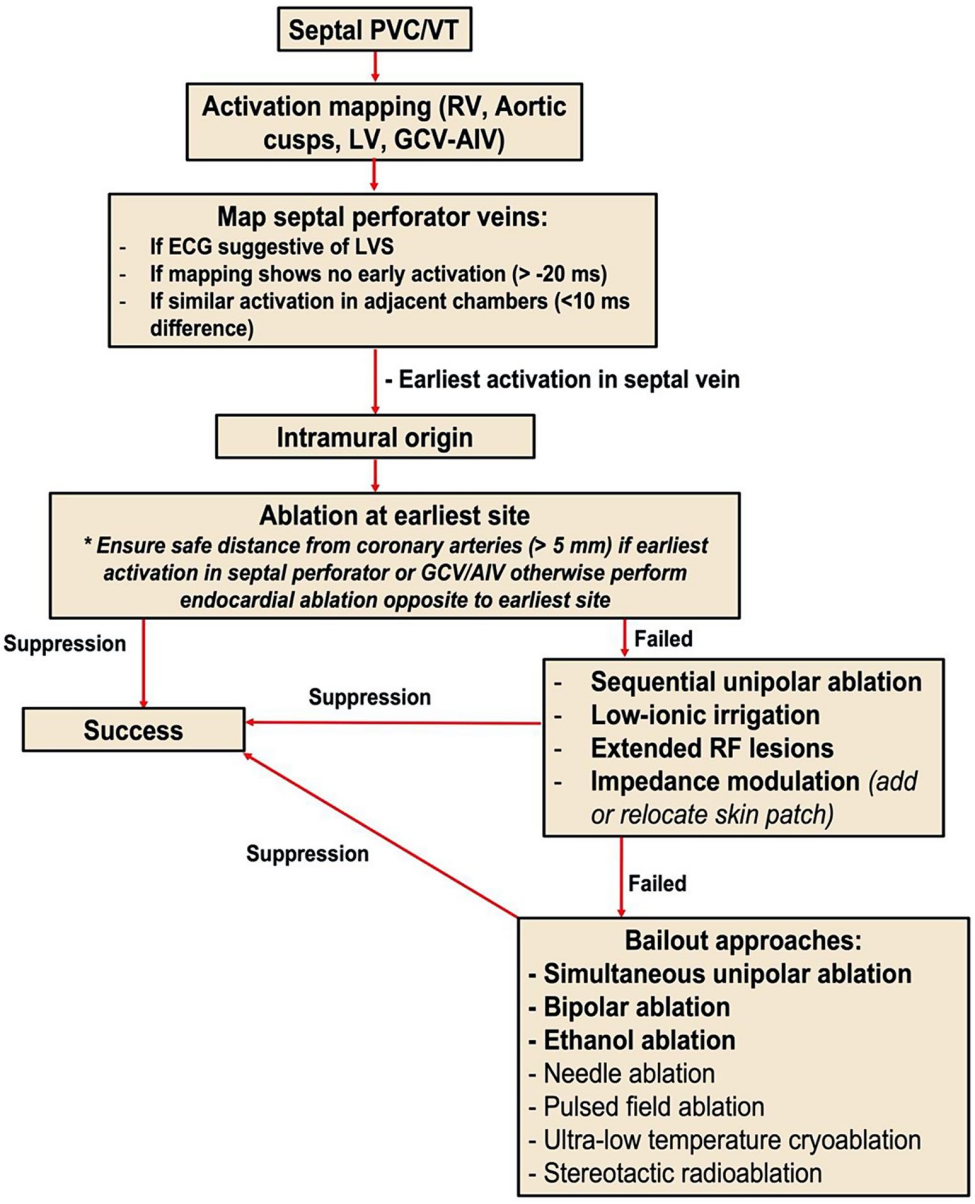
Proposed mapping and ablation algorithm for intramural septal ventricular arrhythmias. PVC = premature ventricular contraction, VT = ventricular tachycardia, RV = right ventricle, LV = left ventricle, GCV = great cardiac vein, AIV = anterior intraventricular vein, RF = radiofrequency

There are no specific clinical or electrocardiographic (ECG) features to reliably predict an intramural VA source. However, several elements increase the pre-test probability and should be considered when planning an ablation procedure (Fig. 1). These include:

- Twelve-lead ECG suggesting an origin from the LV ostium or the interventricular septum: right bundle branch block (RBBB) pattern with inferior axis or left bundle branch block (LBBB) pattern with V2 or V3 transition and either inferior axis, superior axis or inferior lead discordance (positive lead II / negative lead III).
- Nonischemic cardiomyopathy, especially genetic cardiomyopathies such as those associated with pathogenic LMNA and TTN mutations.
- Previous failed endocardial and/or epicardial ablation.
- Pre-procedural imaging (computed tomography or magnetic resonance imaging) demonstrating the presence of intramyocardial scar.

### Diagnosis of Intramural Ventricular Arrhythmia

In focal VT or premature ventricular complexes (PVCs), mapping of the endocardium and epicardium usually provides only indirect clues to an intramural site of origin. For VAs from the interventricular septum, direct access to the intramural space can be achieved by selectively cannulating and mapping the septal branches of the anterior interventricular vein (AIV) (see next section). In general, the following electrophysiological findings suggest a focal intramural origin:

- Earliest ventricular activation in the endocardium or epicardium (directly or via epicardial coronary veins) < 20 ms ahead of the QRS onset.
- Similar activation timing in 2 or more adjacent cardiac chambers (right ventricle [RV], LV, or epicardium) (< 10 ms difference).
- Absence of distinct QS unipolar electrograms at the site of earliest endocardial or epicardial activation.
- Far-field electrograms at the site of earliest endocardial or epicardial activation.
- Suboptimal pace mapping from earliest activation site in the endocardium or epicardium.
- Earliest ventricular activation mapping recorded in a septal venous branch (LV annular vein or septal perforator branches of the AIV).

In reentrant VTs, determination of an intramural isthmus relies on detailed activation and entrainment mapping efforts if the VT is inducible and hemodynamically tolerated. First, it is always useful to confirm a reentrant mechanism by demonstrating progressive fusion with RV pacing at different cycle-lengths. On activation mapping, the main criterion for an intramural isthmus is an incomplete recording of the diastolic activation, with evidence of activation gaps on both endocardium and epicardium. If the mapping window is annotated to the onset of the QRS and divided in 8 isochrones, the yellow and green isochrones corresponding to the mid-isthmus activation will be typically missing from the map. Depending on the extent of the VT isthmus lying in the intramyocardium, activation may look focal on the endocardial map, epicardial map or both. Additionally, overdrive pacing from the endocardium and epicardium will reveal only manifest fusion, consistent with bystander or outer loop components, highlighting lack of access to the isthmus. If the VT is not tolerated or noninducible, determination of an intramural isthmus is more difficult and may be suspected when pace mapping for the clinical VT is poor in the endocardium or epicardium or if the VT has septal morphology, but bipolar voltage mapping shows no distinct endocardial scar. In these cases, unipolar voltage mapping may be helpful to identify intramural scar. Finally, non-ischemic septal VT substrate may further be assessed by pacing the RV base and measuring transmural conduction time along the LV septum, with areas exhibiting delayed transmural conduction time (> 40 ms) indicating potential targets for substrate modification [5]. Like focal VAs, septal mapping via septal perforator veins may provide access to intramural substrate for voltage mapping, pace mapping, and entrainment.

## Mapping Approach

Accurate localization of intramural VAs usually requires comprehensive mapping of the adjacent cardiac chambers, ideally with the aid of multipolar mapping catheters. For intramural septal VAs this involves mapping the right and left sides of the interventricular septum, and for VAs from the LV ostium or LV free wall, this is achieved by mapping the LV endocardium and the epicardial surface of the LV via the coronary venous system or directly by percutaneous epicardial access. In focal VT/PVCs, activation time in both surfaces is similar and often diffuse, with suboptimal pace mapping from either side.

**Fig. 2:**
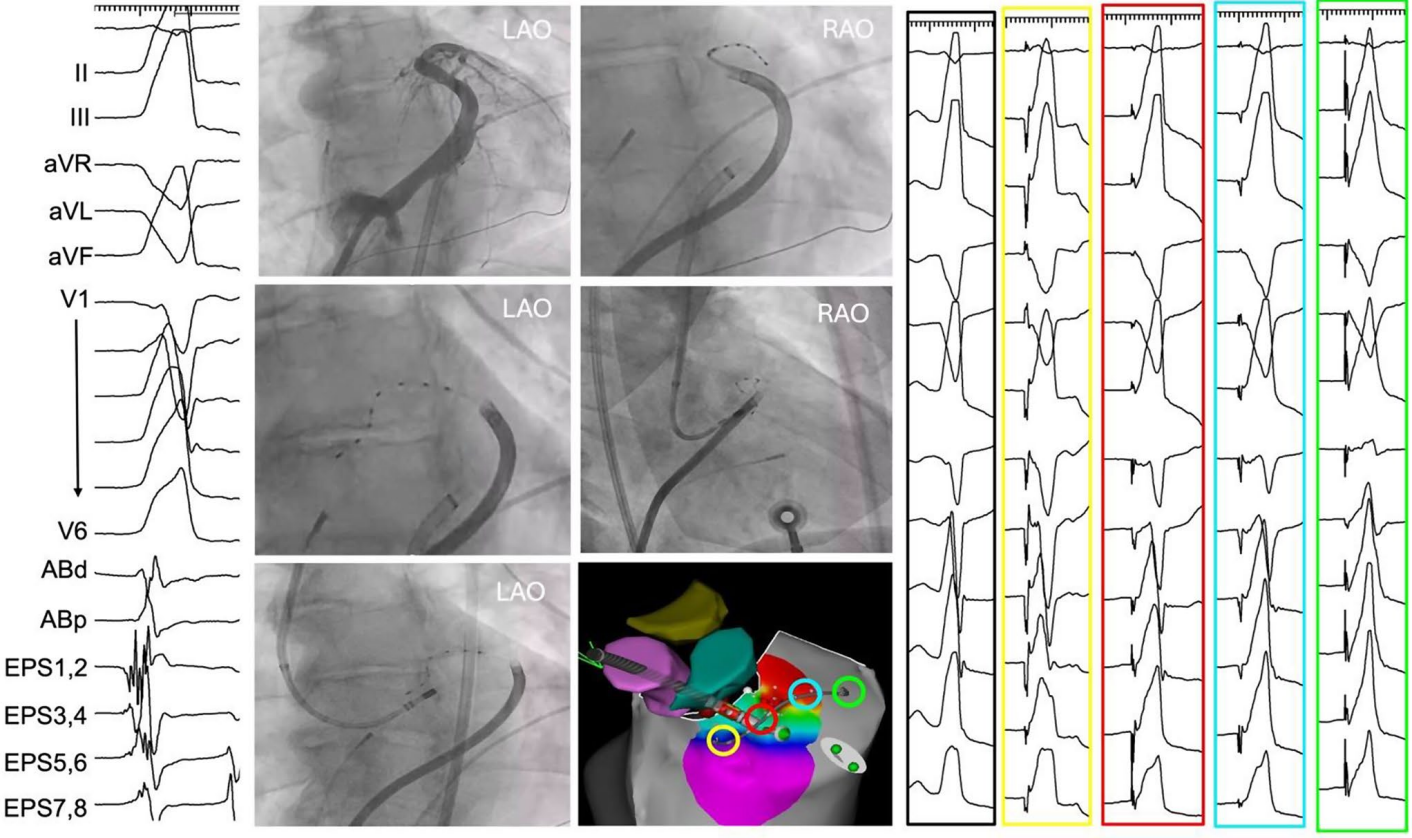
Mapping of intramural outflow tract PVC with earliest activation recorded on a decapolar catheter (EPstar) positioned in the LV annular vein (bipole 3,4), where the best pace mapping was also obtained (red box) (clinical PVC depicted in the black box). A CS venography was performed to define the coronary venous anatomy **(A)**, followed by mapping of the AIV **(B)** and LV annular vein **(C).** Successful suppression of the PVC was achieved by RF application from the LV endocardium, immediately adjacent to the earliest intramural site (**D**, **E**, **F**). PVC = premature ventricular contraction, RF = radiofrequency, LV = left ventricle

As previously mentioned, in case of septal VAs selective mapping of the septal coronary veins offers a unique opportunity to directly explore the intramural aspect of the septum to obtain activation timing information or to perform entrainment maneuvers and/or pace mapping (Fig. 2). Mapping of the septal coronary veins is performed by advancing a deflectable sheath (usually a large curl Agilis sheath, Abbott) into the coronary sinus (CS). A detailed CS venogram should always be performed as there are considerable anatomic variations amongst patients. Once the individual venous anatomy is defined, one or more small multielectrode catheters (Map-iT, Access Point Technologies/Stereotaxis; or EPStar, Baylis) can be advanced through the sheath to the area of interest, sometimes aided with angiography catheters to subselect vessels. Alternatively, mapping can be completed using dedicated mapping wires (Visionwire, Biotronik) or conventional angioplasty wires (Balanced Middleweight guide wire 0.0014”, Abbott) to collect unipolar signals from the venous system. If using an uncoated wire for mapping, the wire should be advanced through a microcatheter leaving only the distal wire tip exposed to collect only local electrograms and avoid a large antenna effect.

**Fig. 3:**
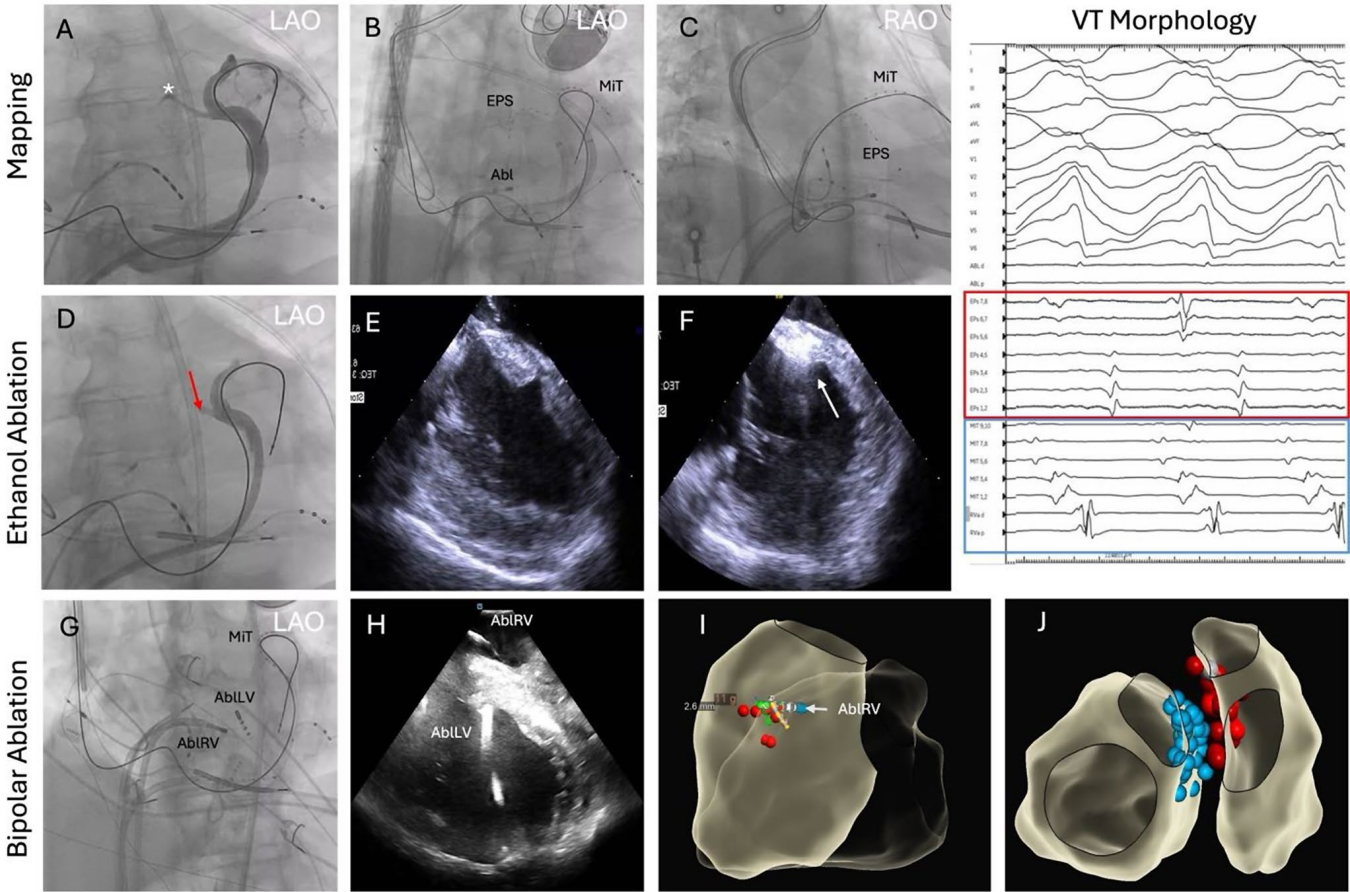
Septal ventricular tachycardia with RBBB pattern and right inferior axis (inset panel). After the anatomy of the coronary venous system was defined with venography **(A)**, a Map-iT (MiT) catheter was placed in the AIV and an EPstar (EPS) catheter was placed in the first septal perforator vein (*) as seen in LAO **(B)** and RAO projections **(C)**. Electrograms from both catheters can be seen on the VT morphology panel during arrhythmia, with mid-diastolic activity recorded by the EPstar catheter (red box). After failed endocardial ablation from both sides of the septum, ethanol ablation through the septal vein was performed (**D**; red arrow), with acute suppression but VT recurrence over the next days. Lesion formation can be seen on ICE with pre- **(E)** and post-ethanol infusion images demonstrating echodense lesion formation (F, white arrow). Ultimately, the patient was taken back to the lab and bipolar ablation was performed with two ablation catheters on opposite sides of the interventricular septum (AblRV and AblLV) as seen on fluoroscopy **(G)**, ICE **(H)**, and electroanatomic map **(I)**. The final lesion set is demonstrated in panel J. AIV = anterior intraventricular vein, LAO = left anterior oblique, RAO = right anterior oblique, VT = ventricular tachycardia, ICE = intracardiac echocardiography

When approaching arrhythmias arising from the LV summit, the authors recommend a triangulation method that involves simultaneous mapping of the endocardium, epicardium, and intramural septum. This is achieved by placing a multielectrode catheter in the great cardiac vein (GCV)/AIV junction (epicardium), a second multielectrode catheter in an LV annular vein or first septal perforator vein (intramural), and an ablation or multipolar mapping catheter in the subaortic LV via retrograde aortic or transseptal access (endocardium). This is useful not only in determining the earliest site but may also guide the site of ablation (i.e. endocardial vs. epicardial from the venous system). A similar approach can be used for LV crux arrhythmias with the ablation catheter placed at the earliest endocardial site and a multielectrode mapping catheter advanced into the middle cardiac vein (MCV). Earliest activation in the MCV would suggest a true crux origin and early endocardial activation would suggest an inferoseptal LV origin.

## Ablation Strategies

In many cases, intramural VAs can be successfully eliminated by conventional radiofrequency (RF) ablation delivered from the closest endocardial site, but prolonged lesions (up to 5 min) or ablation from multiple sites may be necessary. Typical unipolar ablation generates a lesion depth of 5.7 ± 0.4 mm under standard conditions in an ex vivo model [6]. As the myocardium can be > 10 mm thick in many areas including the septum and LV ostium, transmurality is often difficult to achieve. The use of low-ionic irrigant solutions, such as half-normal saline (HNS; 0.45% NaCl) and 5% dextrose in water (D5W), may also enhance current delivery to the tissue [7] and result in increased lesion depth and size [6, 8]. In a study of 94 patients with VAs refractory to standard catheter ablation, irrigation with HNS resulted in acute arrhythmia suppression in 84% of cases with one year VT-free survival of 89.4% [7].

When standard ablation fails or results only in transient arrhythmia suppression, several bailout ablation strategies can be considered:

### Simultaneous Unipolar Ablation

Simultaneously approaching the area of interest from two vantage points may result in deeper lesion formation than sequential unipolar ablation. For simultaneous RF unipolar ablation, catheters are placed on adjacent structures and RF is delivered through both catheters at the same time. This technique requires two open-irrigated catheters attached to two generators, which can increase equipment cost.

### Bipolar Ablation

Bipolar RF ablation is performed by placing ablation catheters at adjacent cardiac structures surrounding the VA focus (Fig. 3). Energy is delivered from one catheter while the second catheter is used as return electrode instead of the dispersive skin patch, with the size of the return catheter electrode modulating lesion characteristics [9]. Bipolar ablation has been shown in ex vivo models to generate deeper lesions when compared to unipolar ablation. Its use has been reported to target refractory VAs in the interventricular septum and LV ostium/LV summit [10–12]. Bipolar ablation may increase the risk of ablation related complications, though with careful power titration and close attention to ablation parameters serious complications can be avoided [10].

### Ethanol Ablation

Ethanol ablation delivered into the coronary venous system is an established therapy for mitral annular flutter and atrial fibrillation (vein of Marshall ablation) and has been increasingly used also for VAs. Ethanol ablation targets for VAs include the septal perforator branches and the annular vein in the LV summit [13, 14]. When arrhythmia substrate is identified in a venous branch, occlusion using angioplasty balloons and infusion of ethanol distal to the balloon can result in dense scar in the myocardium drained by the vein (Fig. 3).

### Pulsed Field Ablation

Pulsed field ablation is a nonthermal ablative modality that uses ultrashort, high-voltage electrical pulses to create microscopic pores in cell membranes leading to apoptotic cell death. An established therapy for the treatment of atrial fibrillation, there is increasing exploration of this technology for the treatment of VAs [15–17]. Preclinical work has demonstrated effective transmural lesion formation in ventricular tissue [18]. Ventricular lesions in a swine model were shown to reach a maximum depth of 9.4 mm [18]. Long-term efficacy and safety profile of this technology remain an area of active investigation.

### Ultra-Low Temperature Cryoablation (ULTC)

This novel ablation strategy uses liquid nitrogen cooled catheters capable of reaching − 196 °C, and preclinical trials have demonstrated transmural lesion formation in atrial and ventricular tissue [19]. In the first in-human trial including 13 patients with monomorphic VT, ULTC resulted in acute arrhythmia non-inducibility in 91% of patients [20]. Long-term safety and efficacy of this technology needs further evaluation.

### Needle Ablation

Needle ablation uses conventional RF in addition to intramyocardially delivered saline to increase conductive heating of tissue. A specialized catheter is positioned at the site of interest and an extendable needle is advanced up to 8 mm into the tissue [21]. This technique generates intramyocardial lesions with increased lesion size when compared to conventional RF [22]. Studies in patients refractory to conventional catheter ablation show 48% freedom from VAs at 6 months with a further 19% experiencing decreased burden [23]. The complication rate in this 31 patient case series was 23% including pericardial effusion, pulmonary embolism, and LV lead dislodgement [23], highlighting the need for ongoing study.

### Stereotactic Radiation

Stereotactic radiotherapy has been explored as an ablation technique for patients with refractory ventricular tachycardia, unsuccessful catheter ablation with advanced techniques, and those that not candidates for catheter ablation [24, 25]. Typically delivered in a single dose of 25 Gy, it has been shown to be effective for decreasing the burden of VT taking between 1 and 7 weeks to reach full efficacy [24]. At present, the recurrence rate for this therapy remains high and it is reserved for patients refractory to catheter ablation or are unable to undergo ablation, and longer-term effects of the radiation are yet unknown [24, 25].

### Procedural Endpoints

For PVC ablations, acute procedural success implies complete elimination of spontaneous or inducible PVCs after a waiting time of at least 30 min despite isoproterenol infusion.

For VT ablations, the ideal procedural endpoint is the non-inducibility of any sustained monomorphic VT at the end of the procedure with programmed electrical stimulation. Induction of a nonclinical VT is considered partial success and may be an acceptable outcome if the clinical VTs have been successfully targeted. When VT is not inducible at baseline, surrogate endpoints should be defined, such as elimination of late potentials, ablation of all local abnormal ventricular activities (LAVAs) or core isolation of the arrhythmogenic scar.

## Conclusion

An intramural location of the arrhythmogenic tissue is an important reason of failed VT/PVC ablation. Mapping of the endocardium and epicardium only provides indirect clues to an intramural origin, and ablation using standard thermal energies has limited ability to penetrate deep tissues in the ventricular wall. Different ablation strategies can be employed in these cases to enhance lesion depth and improve ablation outcomes.

### Key References

1. G. S. Guandalini et al., “Intramyocardial mapping of ventricular premature depolarizations via septal venous perforators: Differentiating the superior intraseptal region from left ventricular summit origins,” *Heart Rhythm*, vol 19, no. 9, pp. 1475-1483 March, 2022, 10.1016/j.hrthm.2022.03.004.

Detailed venous system mapping allows increased recognition of arrhythmia site of origin and impacts ablation strategy.

2. A. Enriquez et al., “Bipolar ablation involving coronary venous system for refractory left ventricular summit arrhythmias,” *Heart Rhythm O2*, vol. 5, no. 1, pp. 24–33, Jan. 2024, 10.1016/j.hroo.2023.11.015.

Bipolar ablation is a safe and effective therapy for ventricular arrhythmias refractory to endocardial ablation.

## Data Availability

No datasets were generated or analysed during the current study.
